# Enhancing diabetic retinopathy diagnosis: automatic segmentation of hyperreflective foci in OCT via deep learning

**DOI:** 10.1007/s10792-025-03439-z

**Published:** 2025-02-18

**Authors:** Yixiao Li, Boyu Yu, Mingwei Si, Mengyao Yang, Wenxuan Cui, Yi Zhou, Shujun Fu, Hong Wang, Xuya Liu, Han Zhang

**Affiliations:** 1 Department of Ophthalmology, Shandong Provincial Hospital, Cheeloo College of Medicine, Shandong University, Jinan, 250012 Shandong Province China; 2 Chang Guang Satellite Technology Co. Ltd, Changchun, 130102 Jilin Province China; 3 Department of Ophthalmology, Qilu Hospital of Shandong University, Jinan, 250012 Shandong Province China; 4 School of Mathematics, Shandong University, Jinan, 250100 Shandong Province China; 5 School of Computer Science and Technology, Shandong Jianzhu University, Jinan, 250101 Shandong Province China; 6 Department of Ophthalmology, Xuzhou Medical University, Affiliated Hospital of Xuzhou Medical University, Xuzhou, 221002 Jiangsu Province China

**Keywords:** Hyperreflective foci, Deep learning, Segmentation algorithm, Diabetic retinopathy

## Abstract

**Objective:**

Hyperreflective foci (HRF) are small, punctate lesions ranging from 20 to 50 $\mu$m and exhibiting high reflective intensity in optical coherence tomography (OCT) images of patients with diabetic retinopathy (DR). The purpose of the model proposed in this paper is to precisely identify and segment the HRF in OCT images of patients with DR. This method is essential for assisting ophthalmologists in the early diagnosis and assessing the effectiveness of treatment and prognosis. In this study, we introduce an HRF segmentation algorithm based on KiU-Net, the algorithm that comprises the Kite-Net branch using up-sampling coding to collect more detailed information and a three-layer U-Net branch to extract high-level semantic information. To enhance the capacity of a single-branch network, we also design a cross-attention block (CAB) which combines the information extracted from two branches. The experimental results demonstrate that the number of parameters of our model is significantly reduced, and the sensitivity (SE) and the dice similarity coefficient (DSC) are respectively improved to 72.90$\%$ and 66.84$\%$. Considering the SE and precision(P) of the segmentation, as well as the recall ratio and recall P of HRF, we believe that this model outperforms most advanced medical image segmentation algorithms and significantly relieves the strain on ophthalmologists.

**Purpose:**

Hyperreflective foci (HRF) are small, punctate lesions ranging from 20 to 50 μm with high reflective intensity in optical coherence tomography (OCT) images of patients with diabetic retinopathy (DR). This study aims to develop a model that precisely identifies and segments HRF in OCT images of DR patients. Accurate segmentation of HRF is essential for assisting ophthalmologists in early diagnosis and in assessing the effectiveness of treatment and prognosis.

**Methods:**

We introduce an HRF segmentation algorithm based on the KiU-Net architecture. The model comprises two branches: a Kite-Net branch that uses up-sampling coding to capture detailed information, and a three-layer U-Net branch that extracts high-level semantic information. To enhance the capacity of the network, we designed a cross-attention block (CAB) that combines the information extracted from both branches, effectively integrating detail and semantic features.

**Results:**

Experimental results demonstrate that our model significantly reduces the number of parameters while improving performance. The sensitivity (SE) and Dice Similarity Coefficient (DSC) of our model are improved to 72.90% and 66.84%, respectively. Considering the SE and precision (P) of the segmentation, as well as the recall ratio and precision of HRF detection, our model outperforms most advanced medical image segmentation algorithms

**Conclusion:**

The proposed HRF segmentation algorithm effectively identifies and segments HRF in OCT images of DR patients, outperforming existing methods. This advancement can significantly alleviate the burden on ophthalmologists by aiding in early diagnosis and treatment evaluation, ultimately improving patient outcomes.

## Introduction

   Diabetes mellitus, a global epidemic, gives rise to a range of macrovascular and microvascular complications. The number of patients with diabetes is expected to increase to 643 million in the 2030 s, and is projected to affect 693 million adults by 2045 [1]. The soaring prevalence of diabetes inevitably leads to a rapid increase in the incidence of its complications. DR, one of the specific complications of diabetes mellitus, is a progressive dysfunction of the retinal vasculature. DR accounts for a substantial part of vision loss in adults aged 20-74 years, especially in developed countries [2]. Visual impairment caused by DR is irreversible. However, DR is preventable, early diagnosis and regular eye check-ups for DR patients can alleviate the symptoms and prevent the vision loss and blindness [3, 4].

OCT, a non-invasive, non-contact, and high-resolution imaging technology, has become a regular check for diagnosis and a common method to evaluate the prognosis of patients with DR [5]. In OCT images, HRF, which exhibits equal or greater reflectivity compared to retina pigment epithelium (RPE) cells, are identified as distinct, well-defined dot-shaped lesions, ranging from 20 to 50 $\mu$m in diameter and lacking back shadowing [6, 7]. In 2009, Coscas [8, 9] et al. first came up with the concept of hyperreflective dots(HRD) during the checks for patients with age-related macular degeneration(ARMD), and renamed it as HRF in the following references. Nevertheless, over a decade and a half, researchers have formulated totally different hypotheses about its origin. Some suggested that HRF are lipoproteins originating from the breakdown of the blood-retina barrier, but others thought that HRF can be activated microglia, a degenerative process of photoreceptor cells, or even the activated RPE cells migrating to the inner layers of the retina [9–14]. Even though the cause and mechanism of its formation are not clearly stated, numerous studies on DR have found that the increase of the number of HRF is closely associated with the aggravation of the disease [10, 15, 16].

In recent years, the number of HRF has been considered as a key OCT biomarker for the early diagnosis, treatment response, and prognosis of several ophthalmic diseases, including diabetic macular edema(DME), ARMD, and retinal vascular occlusions. However, most clinical studies calculate the number of HRF by manual annotation. By this means, reading OCT images constantly not only consumes the time and energy but also requires doctor to be experienced. Accordingly, setting up an algorithm to automatically segment HRF in OCT images is necessary for providing quantitative analysis, lightening the workload of ophthalmologists, and improving the efficiency of diagnosis, treatment, and the evaluation of prognosis.

Researchers started to study the method to detect and segment the HRF from the perspective of digital image processing until 2017. Marzieh Mokhtari et al. [17] constructed the first automatic detection method of HRF based on Morphological Component Analysis (MCA). In 2018, Idowu Paul Okuwobi et al. [18] segmented HRF in 3D OCT images based on region-growing algorithm, helping the mean DSC attain 63.80$\%$ and opening a new gate of segmentation of HRF by computer. In 2019, Idowu Paul Okuwobi et al. [19] established a new segmentation algorithm based on morphological reconstruction and component tree, making the mean DSC reach 70.10$\%$.

Later, researchers began to use deep-learning for the segmentation. In 2018, Thomas Schlegl et al. [20] established an automatic segmentation method, on the basis of residual U-Net, for HRF of patients with neovascular Age-related Macular Degeneration (nAMD) and DR. This algorithm is the first one to use deep-learning for HRF segmentation, and its mean DSC reaches 64.35$\%$. Subsequently, researchers [21–23] constantly ameliorated the algorithm of segmentation and the accuracy increased accordingly. Laszlo Varga et al. [24] applied different neural networks for segmentation. On the one hand, CNN is used to segment the entire image. On the other hand, the image-extracted features are input into another network for pixel-level classification. Md Zahangir Alom et al. [25] proposed R2U-Net, which core innovation lies in its incorporation of Recurrent Residual Convolutional Layers (RCLs) within the U-Net architecture. These RCLs enhance performance in segmentation tasks by effectively accumulating features and providing a more robust feature representation. In 2021, Yao Chenpu et al. [26] proposed a self-adaptive network(SANet) for HRF segmentation, making the mean DSC reach 73.69$\%$, better than U-Net. Later, Yao Chenpu et al. [27]conducted further research on HRF segmentation and proposed a novel global information fusion and dual decoder collaboration-based network(GD-Net), which divided HRF into hard exudate(HE) and microglia for joint segmentation. This method is the first joint segmentation algorithm to distinguish the HE and the microglia, providing a new idea for HRF segmentation. Later, researchers began to combine Transformer with CNN. Zhang et al. [28] proposed TransFuse, which combines Transformers and CNNs in a parallel manner, effectively capturing both global dependencies and low-level spatial details in a more straightforward way. Furthermore, it introduces a novel fusion technique?the BiFusion module?to efficiently fuse multi-level features from both branches. Chen J et al. [29] proposed TransUNet, which utilizes a Transformer with a self-attention mechanism to encode tokenized image patches derived from CNN feature maps into an input sequence for extracting global context. To compensate for the loss of feature resolution caused by the Transformer encoder, TransUNet employs a hybrid CNN-Transformer architecture. The decoder upsamples the encoded features and combines them with different high-resolution CNN feature maps skipped from the encoder path to enable precise localization. TransUNet achieved superior performances competing methods on different applications including multi-organ cardiac Code models.

Compared with other network structures, U-Net [30] pays more attention to the high-level semantic information extracted from the deep layer of the encoder, and ignores the underlying detailed information, due to the process of increasing the receptive field by constant down-sampling in the coding stage. Jeya Maria Jose Valanarasu et al. proposed KiU-Net [31]. This network, focusing on the smaller and more detailed information in the image, first designs an over-complete network Kite-Net. KiU-Net changes the down-sampling coding method of the original U-Net and adopts up-sampling for coding, focusing on the smaller and detailed information in the image. In addition, the Cross Residual Feature Block (CRFB) is designed in KiU-Net to combine Kite-Net and U-Net to form KiU-Net, which supplements the subtle structure ignored in U-Net. Experimental results show that KiU-Net can significantly improve the effectiveness of small and subtle structures in medical segmentation.

In this paper, we propose an HRF segmentation network model based on KiU-Net. This model combines a U-Net and an over-complete Kite-Net. Kite-Net replaces the pooling layer with upsampling layer to extract more detailed information. Compared with the heavy calculation amount by three layers of Kite-Net in the KiU-Net, this model only adopts two layers of Kite-Net to reduce the calculation load and ensure the usefully extracted information for the supplement of U-Net. In addition, this model designs a CAB that combines the detailed information extracted from Kite-Net with the high-level semantic information of U-Net, improving the feature learning ability of two branch networks. Compared with KiU-Net, our model can not only reduce the computational cost and training time but also segment the HRF more accurately. This HRF automatic segmentation model can significantly alleviate the load of ophthalmologists and provide great assistance for the early diagnosis, the evaluation of treatment, and the prognosis of retinal diseases.

## Methods

### Data acquisition

**Fig. 1:**
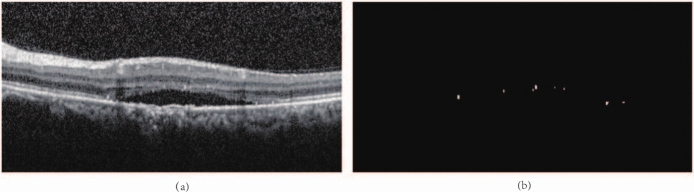
The results of manually annotated images

The dataset includes 173 OCT images of 50 patients, which were obtained from the Department of Ophthalmology, Qilu hospital affiliated with Shandong University, China, and were all approved by the Medical Ethics Committee of Qilu Hospital, China. These images involves DR in different stages. All OCT images of those patients were acquired by using a Heidelberg OCT scanner, and diagnosed by experienced ophthalmologists. Two experts of Ophthalmology carefully examined 173 SD-OCT images, and the annotated sections with HRF. The initial resolution of each slice was 350 $\times$ 700, and the image resolution was resized to 256 $\times$ 512 pixels during the experiment by using MATLAB(2020b). This resizing process was implemented to standardize the input data, ensuring uniformity across all images for subsequent analysis. Figure 1 shows OCT slices with HRF and corresponding manually annotated binary maps.

**Fig. 2:**
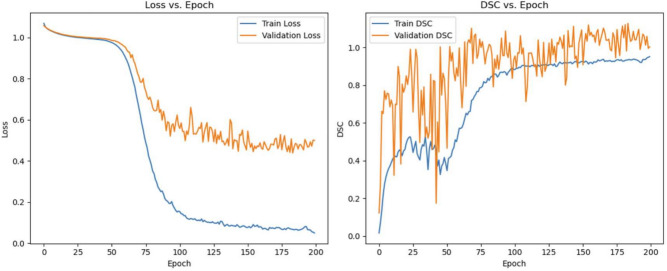
Epoch plot. Both loss and DSC tend to be stable after 150 epochs

All the 173 OCT images from 50 patients were divided into the training set, validation set and test set, according to the ratio of 8:1:1, namely 138 images for training set, 18 images for validation set and 17 images for test set. This experiment program was written in Python and built via the Pytorch framework. The training and testing process is completed on a GPU server with Nvidia RTX 3090 graphics card. The training parameters were set as follows: We used the Adam optimizer, with the momentum parameter B1 as 0.9, and B2 as 0.999. The initial learning rate was 0.0001, with a total of 150 epochs(Fig. 2), and the training batch size was set as 1. Since the model’s performance metrics, including loss and DSC, stabilized after 150 epochs, and the performance on the validation set showed no further improvement, we selected 150 epochs for training. If the loss function did not decrease within 5 rounds, the learning rate would be reduced to half of the original. In addition, the experiment used the early stop strategy to prevent the over-fitting phenomenon. Figure 2 shows the epoch plots of train and validation.

### Image preprocessing

OCT images are inevitably influenced by speckle noise, which greatly affects the image clarity [32], due to the intervention of the internal electronic imaging equipment and the external environmental light source. We use the bilateral filtering algorithm to denoise the image.

In the training of neural networks, only by enriching the train data can the model generalization ability be guaranteed and the overfitting phenomenon be avoided. However, lack of data is a common problem in medical images. It’s necessary to take measures to increase the number of samples. In order not to change the lateral features of the retinal layer structure and preserve the original positional feature information, we adopt enhancement methods in this paper including horizontal flipping, random cutting, and random rotation between $0^{\circ }$ and $30^{\circ }$.

### Network architecture design

**Fig. 3:**
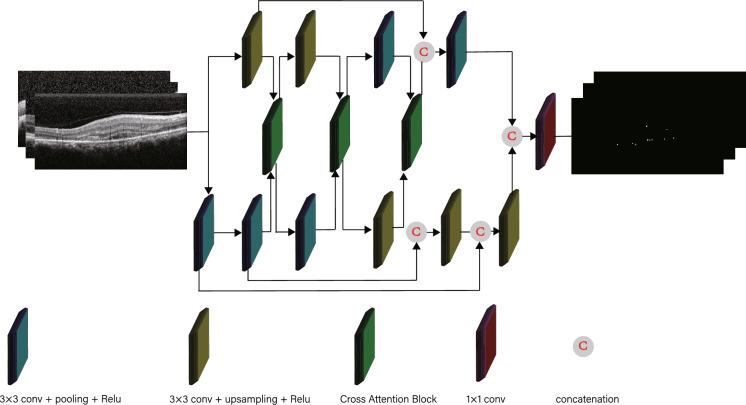
Structure of our model

In order to improve the segmentation performance aiming at the details, Jeya Maria Jose Valanarasu et al [31] designed an over-complete network structure Kite-Net. In the encoder stage, bilinear up-sampling is used to replace the pooling down-sampling of the original U-Net, to sample the input image to a higher resolution and to continuously reduce the receptive field. By this means, more detailed information can be obtained. Then, the KiU-Net is constructed by combining three-layer Kite-Net with three-layer U-Net through the CRFB. Kite-Net can supplement the details that can not be obtained from U-Net, and focus more on HRF to figure out the problem of incomplete segmentation of the details in medical images by U-Net. Therefore, after image preprocessing of denoising and data enhancement, we propose an HRF segmentation network based on KiU-Net to improve the segmentation accuracy. Figure 3 shows the structure of our model.

#### Resolution enhancement

In U-Net encoder, after each convolutional layer, the maximum pooling layer is used to downsample the feature map of this layer, making the input image reflected in a lower spatial dimension. This structure makes the receptive field of the deep filter increase and the deeper network focus on remarkable features, resulting in features which are used to segment small lesions or elaborate edges cannot be extracted. In contrast, at the end of encoder of the Kite-Net, the bilinear up-sampling layer is used to replace the maximum pooling down-sampling layer, giving the input image a higher spatial dimension and focusing more on the features in detail. This can help us segment small targets more efficiently. From the perspective of the space, the encoder of the U-Net is incomplete, making it learn incomplete features and focus more on remarkable features. However, the encoder of the Kite-Net is over-complete [31], limiting the increase of the receptive field and focuses more on detailed features.

In maximum pooling layer of U-Net, set the pooling coefficient as 2 and the step-size as 2. Assuming that the receptive field of the feature map being input into pooling layer is $\kappa$ $\times$ $\kappa$ on the input image, then the receptive field of the feature map after pooling operation changes to 2 $\kappa$ $\times$ 2 $\kappa$. With the increase of the layers, the receptive field gradually expands. Receptive field increase to $2^{2\left( i-1\right) }$ $\kappa$ $\times$ $2^{2\left( i-1\right) }$ $\kappa$. When the pooling layer is replaced by the upsampling layer with a coefficient of 2, the receptive field of the feature map being input into convolutional layer will be $\frac{1}{2}$ $\kappa$ $\times$ $\frac{1}{2}$ $\kappa$, and the receptive field will gradually shrink with the increase of the network layers. The receptive field finally shrinks to $\frac{1}{2}^{2\left( i-1\right) }$ $\kappa$ $\times$ $\frac{1}{2}^{2\left( i-1\right) }$ $\kappa$. This reduction of receptive field is conducive to focusing on local details and improving the segmentation accuracy of detailed structures of HRF.

Kite-Net can extract high-quality detailed features. Nevertheless, because of the lack of filters that can extract high-level semantic information, the performance of using Kite-Net alone is not satisfactory. Accordingly, we combine the Kite-Net and U-Net to improve the feature learning ability. In addition, in order to supplement the details of U-Net branches and not occupy too much of the limited server volume, we combine only two layers of Kite-Net with the bottom two layers of the three-layer U-Net structure to form the improved KiU-Net segmentation network in this paper.

#### CAB model design

To further utilize the capabilities of the two branches, KiU-Net proposed the CRFB to combine the features of the two networks at different scales. Since the features learned by U-Net and Kite-Net are different, CRFB can be used to learn complementary features from the two branches, resulting the further improve of the quality of features learned by individual networks. However, CRFB only performs the simple linear calculation and then adds the features of the two branches. The features extracted by Kite-Net have great influences, making the segmented lesion area blocky and leading inaccurate boundary segmentation. Therefore, we construct a new CAB to combine the characteristics of the two networks at multiple scales.

Assuming that the two branches respectively are branch K and branch U, this CAB module firstly uses the feature map of one of the branches K to guide the generation of the attention figure and then multiplies it with the feature map of the other branch U. The new feature map is obtained and added to the feature map of branch U, which is rated as the residual part. The new output feature map is input into the next layer of branch U. The above-mentioned operations are performed on both branches to further improve the training of a single network.

**Fig. 4:**
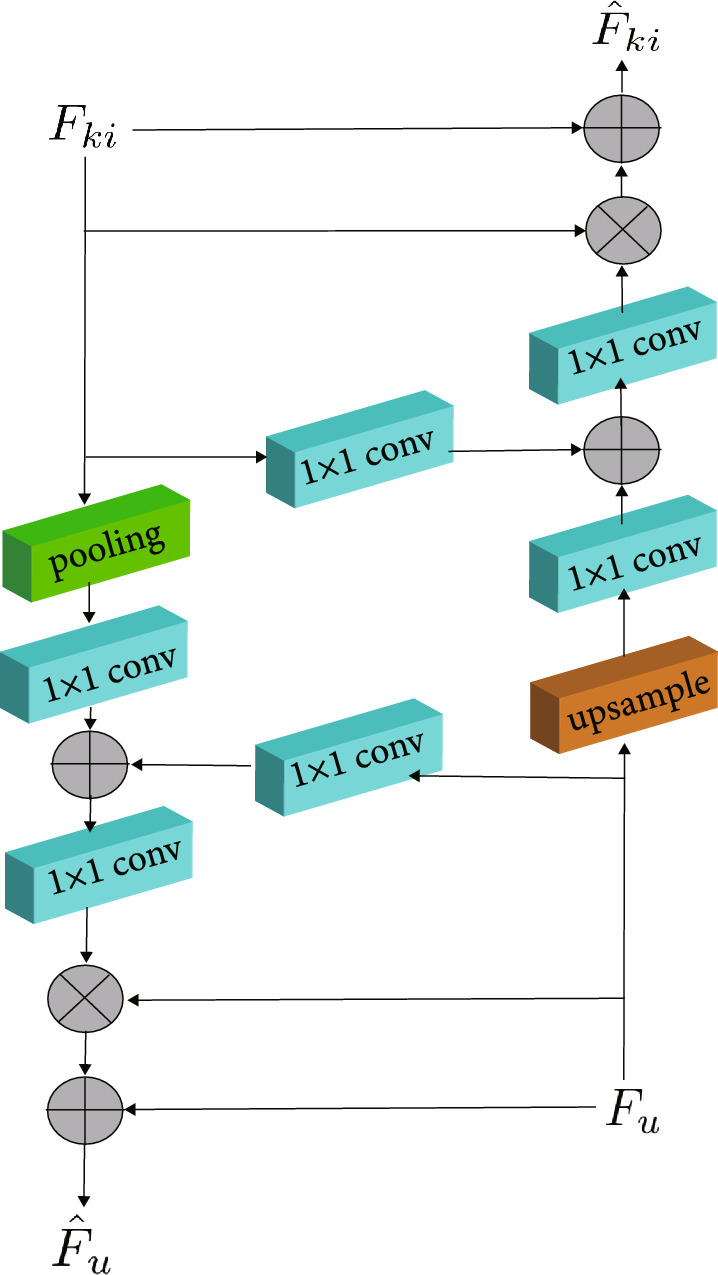
The schematic diagram of the CAB. $F_u$ represents the input of U-Net, and $F_{ki}$ represents the input of Kite-Net

Figure 4 shows the structure of CAB. $F_u$ and $F_{ki}$ have channels with the same number but different spatial resolutions, leading to space between the two dimensions needs to be unified. Downsample the $F_{ki}$ when calculating the output of U-branch Net, to adapt to the $F_u$ size. Subsequently, $F_u$ and downsampled $F_{ki}$ are going to take $1\times 1$ convolution separately, compressing the channel number to the half. Then we add the results after convolution. We activate the above results by using the ReLU function, and once again take the convolution of $1\times 1$, to compress the channel number to 1. After being activated by the sigmoid activation function, the attention figure $\alpha _u$ is obtained. The attention figure $\alpha _u$ multiplies with every channel of $F_u$ and the $F_{ki}$ induced feature map with attention can be obtained. The original feature map $F_u$, which is rated as a residual model, and the feature map mentioned above are combined together to obtain the features of the input $F_u$ as the next layer of the U-Net. This feature not only retains the information of the original map but also combines the information of the Kite-Net, enlarging the weight of location related to the details in $F_u$. The formulas of $F_u$ are as follows:

2.3.1 $$\eqalign{ & {\alpha _u} = {\sigma _2} \times \left( {f \times \left( {{\sigma _1} \times \left( {{f_u} \times ({F_u}} \right)} \right.} \right. \cr & \left. {\left. {\quad \quad + {f_{ki}} \times \left( {down\left( {{F_{ki}}} \right)} \right)} \right)} \right) \cr}$$

2.3.2 $$\begin{aligned} & F_u={\hat{F}}_u + F_u\times a_u \end{aligned}$$

where $f_u$,$f_{ki}$ and *f* are respectively represented for $1\times 1$ convolution targeted at different branches, $\sigma _1$ represents for ReLU activating function, and $\sigma _2$ represents for sigmoid activating function. Likewise, to calculate the feature $F_{ki}$ that will be input to the next layer of Kite-Net, a bilinear interpolation upsample needs to be held on the feature $F_u$ from U-Net, ensuring it has the same spatial dimension as $F_{ki}$. Through the CAB, the information of the two branches can be guided mutually to enhance the performance of a single network. Formulas of $F_{ki}$ are as follows:

2.3.3 $$\begin{aligned}&\alpha _{ki}=\sigma _2\times \left( f\left( \sigma _1\times \left( f_{ki}\times \left( F_{ki}\right) + f_u\times \left( up\left( F_u\right) \right) \right) \right) \right) \end{aligned}$$

2.3.4 $$\begin{aligned}&F_{ki}={\hat{F}}_{ki} + F_{ki}\times a_{ki} \end{aligned}$$

### Evaluation index

Image segmentation is a pixel-level binary classification task, namely, dividing all the pixels in the image into positive groups and negative groups. The pixels in the HRF area belong to positive groups and the pixels in the non-HRF area belong to negative groups. We divide the results of the estimation into four groups: (1) True positive(TP), the number of pixels judged as true are true in fact. (2) False positive(FP), the number of pixels judged as true are false in fact. (3) False negative(FN), the number of pixels judged as false are true in fact. According to the number of pixels that above-mentioned, we calculate the evaluation index of the effect of the segmentation.

In this paper, we use SE, P, and DSC to evaluate the performance of the network. SE is to calculate the proportion of the TP in all positive pixels of real images, reflecting the missing segmentation. In other words, better SE means a fewer rate of missing segmentation. P is to calculate the proportion of the TP in all positive pixels in predicted images, reflecting the erroneous segmentation. In other words, better P means a fewer rate of erroneous segmentation. DSC is to calculate the similarity of two samples, considering SE and P at the same time. A higher DSC means a better effect of segmentation. DSC can be formulated as follows:

3.2.1 $$\begin{aligned} & D S C=\frac{|\textrm{X} \cap \textrm{Y}|}{|\textrm{X}|+|\textrm{Y}|}=\frac{2 T P}{2 T P+F P+F N} \end{aligned}$$

In this paper, we evaluate the performance of the network in two ways: the first one is to calculate the P of lesion segmentation in each pixel through the evaluation index above-mentioned. The other one is that the HRF is correctly predicted if the number of pixels in a certain HRF area of predicted results covered 60$\%$ of pixels in the same area of the real images. According to the index above-mentioned, we calculate the number of HRF being correctly predicted, the number of HRF being erroneously predicted, and the number of HRF that are not predicted, to evaluate the P of the HRF recognition and segmentation of the network, respectively.

### Loss function

Network training commonly use the cross-entropy loss function to compute parameters. However, due to the foreground and background pixels of the HRF images existing serious unbalanced category, the cross-entropy loss function makes the foreground pixels submerged by background pixels, making the forecast results inappropriate and even the predicting figure only has zero value. Focal loss settled this problem by setting a parameter to reduce the influence of easily classifiable samples on the loss function and increase the influence of difficult classifiable samples [33]. The specific calculation formula is as follows:

2.4.1 $$\begin{aligned} & focal\ loss=-\alpha \times \left( 1-p_i\right) ^\gamma \times log\left( p_i\right) \end{aligned}$$

Where $\alpha$ can balance the effect of the positive and negative samples on the loss function, and setting $\gamma$ can balance the impact of the difficult and easy samples on the loss function. Then, we would enhance the weight of hard-to-segment samples and the target sample, and solve the categories imbalances of HRF segmentation. Dice loss belongs to the region-based loss function. It is a measurement function of set similarity [34] and can be used to measure the similarity between the predicted results and the real results. The formula is as follows:

2.4.2 $$\begin{aligned} & dice\ loss =1-\frac{|\textrm{X} \cap \textrm{Y}|}{|\textrm{X}|+|\textrm{Y}|} \end{aligned}$$

where *X* represents the real image segmentation, *Y* represents the predicted segmentation result, ${|\textrm{X} \cap \textrm{Y}|}$ is the intersection between predicted results and actual results, $|\textrm{X}|$ and $|\textrm{Y}|$ respectively represent the positive pixels for *X* and *Y*. Dice loss can effectively solve the problem of uneven positive and negative sample categories. However, once the prediction of some pixels in the target area is wrong, the value of the loss function will be greatly changed, and the gradient will greatly oscillate. Accordingly, the focal loss combined with dice loss is adopted as the loss function in this paper. The formula is as follows:

2.4.3 $$\begin{aligned} & loss=0.5\times dice\ loss +0.5\times focal\ loss \end{aligned}$$

Dice loss can supervise the network for learning via the similarity between predicted results and real results, and Focal loss can guide the Dice Loss and give it a learning direction. The combination of these loss functions is more suitable for the HRF segmentation of small sample objects with an imbalance of positive and negative samples.

## Experimental results and analysis

### Segmentation results between the original images and denoised images

**Fig. 5:**
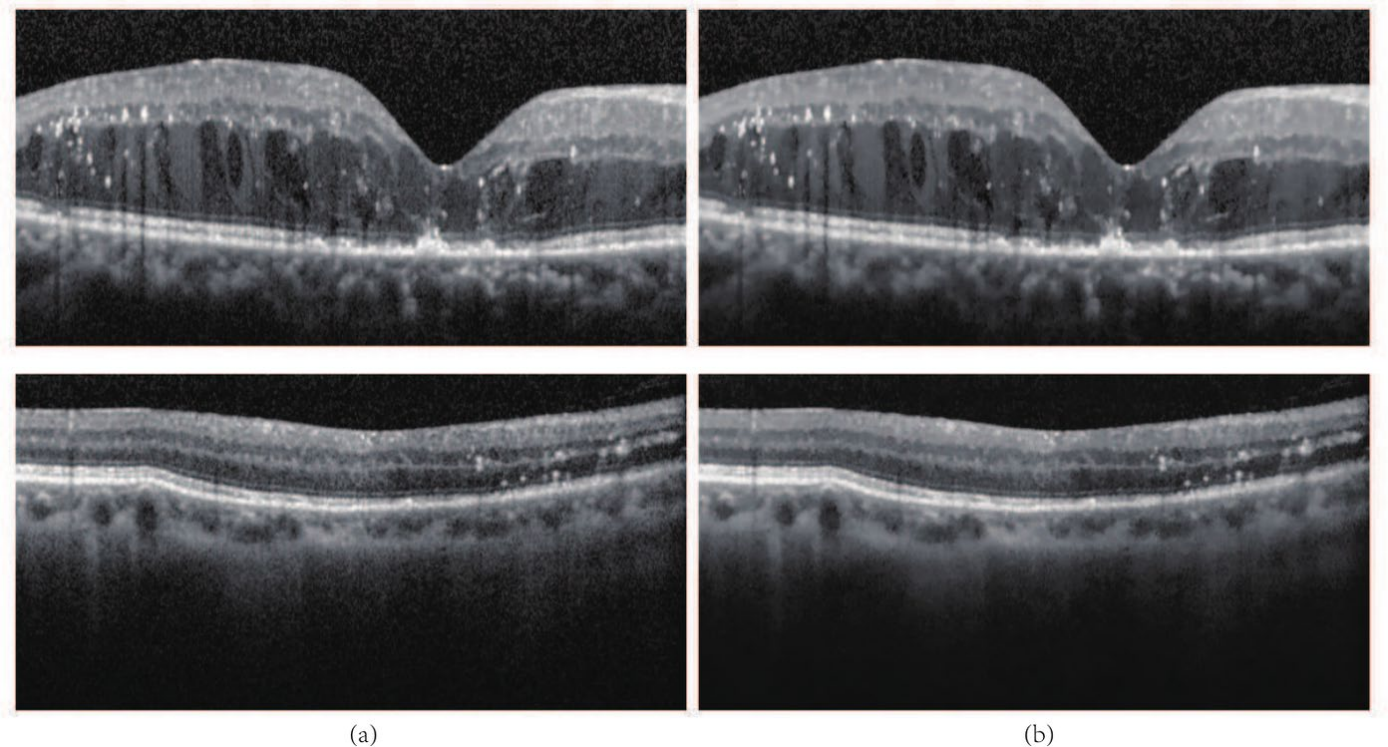
The results of denoising by the bilateral filtering, **a** are the original images and **b** are the images after denoising

Figure 5 illustrates the denoising results. Table 1 presents the segmentation performance of various models before and after denoising. Bilateral filtering demonstrably mitigates speckle noise in OCT images, reducing its impact on image quality and subsequent segmentation tasks without compromising detail preservation. The retinal layer structure appears smoother in the denoised images, while details within the retinal layers and the HRF region are retained. Furthermore, denoising leads to an observable improvement in the segmentation performance across all models.

From Fig. 5, it can be observed that the bilateral filtering denoising algorithm can effectively remove speckle noise in OCT images. At the same time, the details of layer structure and HRF area in denoising image are preserved well, reducing the influence of noise on the quality of images and subsequent segmentation tasks.

**Table 1:** The segmentation index comparison among the different networks before and after denoising

Network architecture	Original images			Images after filtering		
	SE	P	DSC	SE	P	DSC
U-Net	59.72	62.66	57.99	63.49	62.43	60.04
U-Net++	63.17	62.94	61.37	66.78	63.68	62.32
Attention U-Net	66.02	63.24	62.90	67.72	64.25	64.02
SA Net	63.45	63.79	61.56	66.79	63.65	61.88
TransUNet	66.36	64.03	63.20	67.06	64.87	64.30
Proposed Model	67.08	61.83	63.38	70.73	62.59	65.86

### Ameliorated KiU-Net segments HRF with high SE and P

We randomly selected 173 OCT slice images from the data set as the test set. The training set(138 images) is preprocessed and input into the network model for training, and then the performance of the network model is examined using the test set. The average value of the evaluation index of all image segmentation results in the test set are calculated, the SE is 72.90$\%$, P is 66.12$\%$, and DSC is 66.84$\%$, respectively. According to the above evaluation index, the SE of our proposed algorithm could achieve relatively accurate result. According to the evaluation index described in section 2.5, we calculated the number of HRF. The SE is 73.57$\%$, P is 70.70$\%$, and DSC is 71.32$\%$, respectively. From the perspective of the evaluation index of the number of HRF, the results are relatively accurate if only P of the recognition of HRF is considered.

**Fig. 6:**
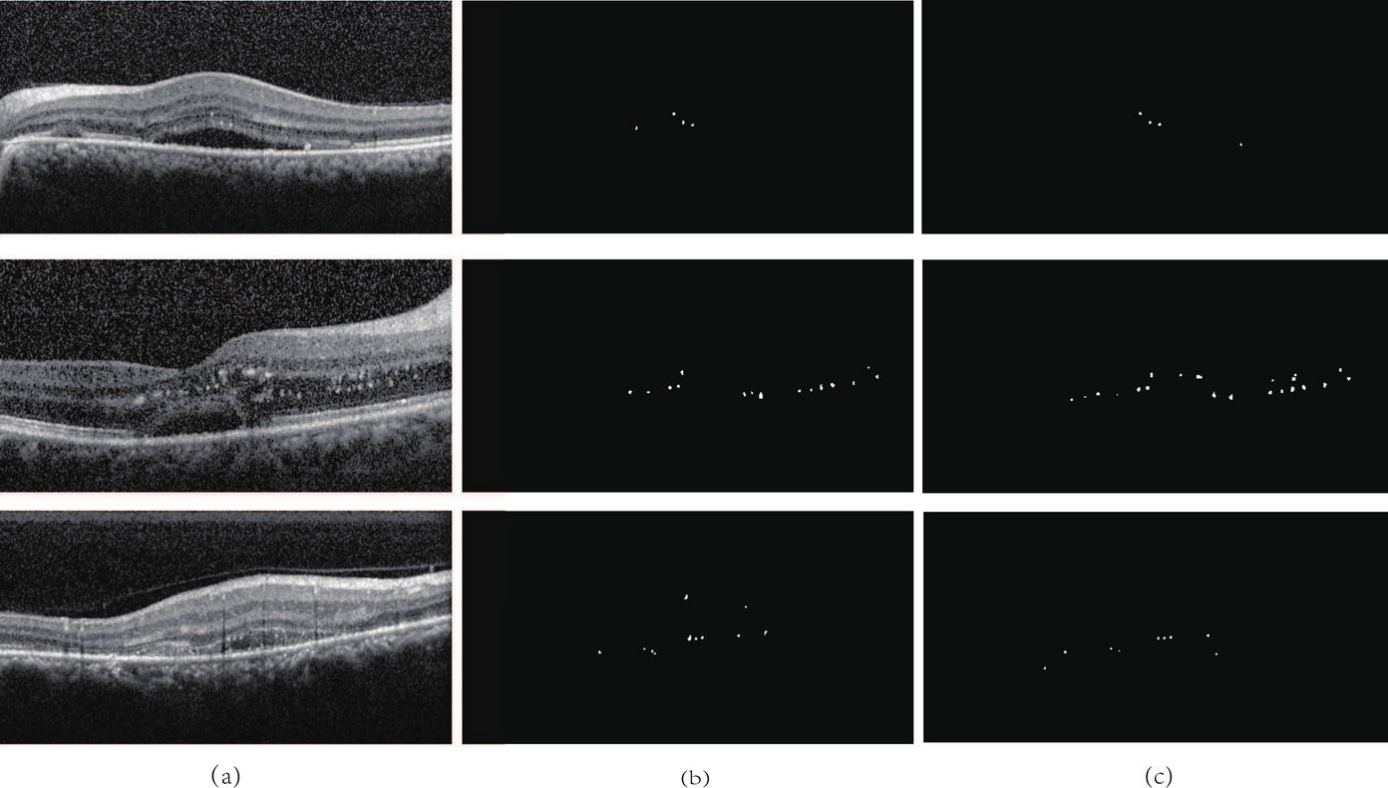
The comparison among the original images, manual segmentation, and results obtained by our proposed algorithm, where **a** are original images in the test set, **b** are manual annotation results, and **c** are results of the algorithm in our model

Figure 6 shows the segmentation results of our proposed model. It is apparent that the segmentation results are similar to the manually annotated results, most HRF can be accurately recognized and segmented, even for OCT images with low-contrast areas and complex lesions.

**Fig. 7:**
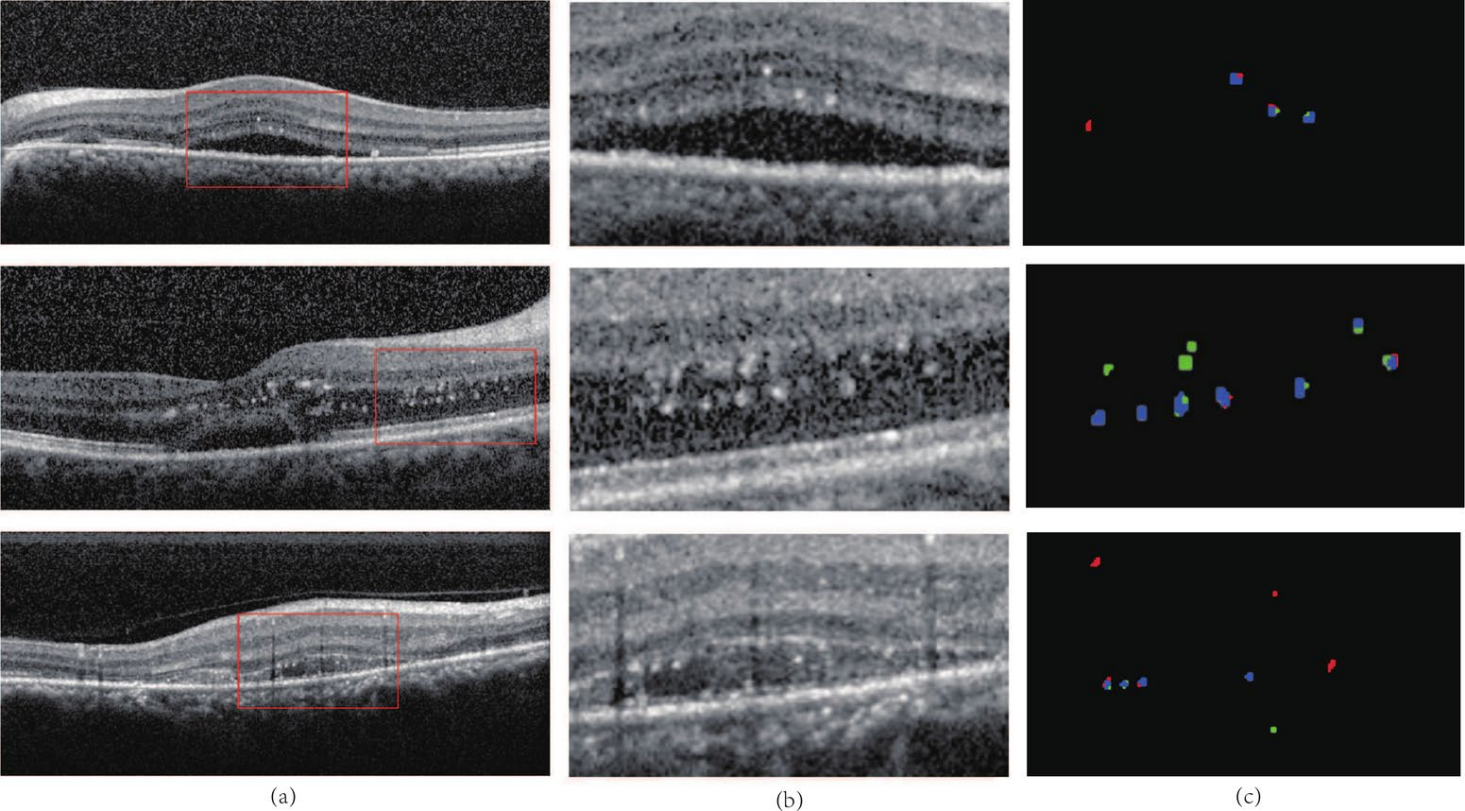
Locally enlarged view of segmentation results, where blue is TP, green is FP, red is FN. The red boxes in **a** are the amplified part. Images of **b** are the enlarged original images and **c** are the results from the comparison between the manual annotation and segmentation

   More detailed comparisons about the locally enlarged segmentation results are presented in the closeups as shown in Fig. 7. In the (c) images, the blue pixel points are the correctly segmented lesions, the red pixel points are unsegmented lesions, and the green pixel points are the points that are erroneously segmented to HRF.

In addition to the correctly segmented regions, there are many green regions in the segmentation results. These green regions can be mainly divided into two categories. The first category is located at the edge of the HRF region, indicating that our model tends to segment larger HRF boundaries in contrast to the manually annotated HRF. The second category is that the areas with locally high contrast can be easily recognized as HRF by this algorithm, leading to erroneous segmentation of these lesions that are not HRF. The missing segmentation area is mainly due to the fact that there is a strip area with lower gray level similar to the artifact below HE under the lesion area, and the network learned this feature, so it does not think that the lesion should be rated as the target area.

### Ameliorated KiU-Net performs well compared with other five methods

To illustrate the superiority of the network model proposed in this paper, we compared it with following networks: U-Net [30], the U-Net ++ [35], Attention U-Net [36], the SANet [26], and TransUNet [29].

**Fig. 8:**
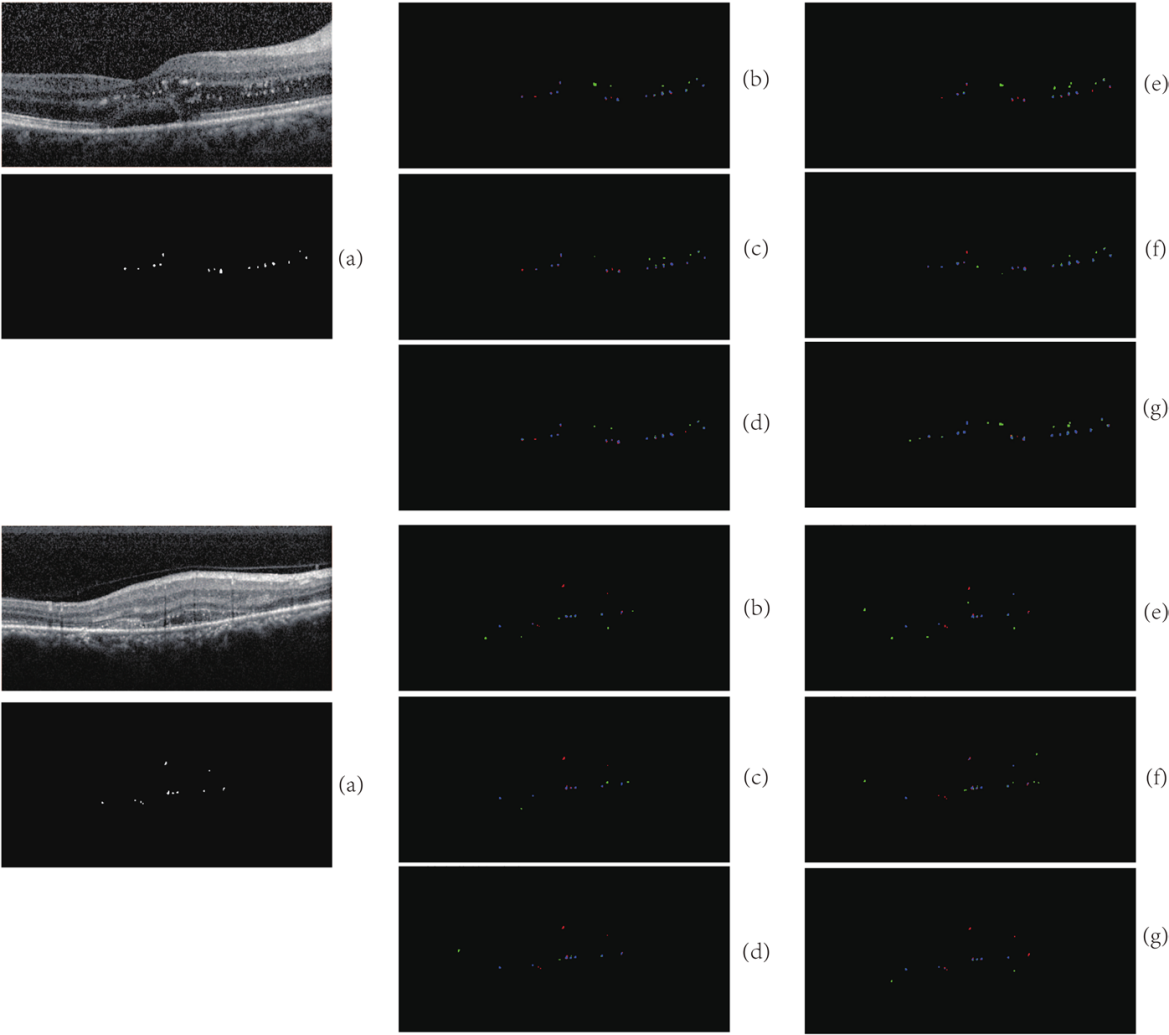
The results of segmentation among different networks. Blue is TP, green is FP, red is FN. **a** are original images. **b** are images of manually annotation. **c** are images of U-Net. **d** are images of U-Net++. **e** are images of Attention U-Net. **f** are images of SANet. **g** are images of our model

**Fig. 9:**
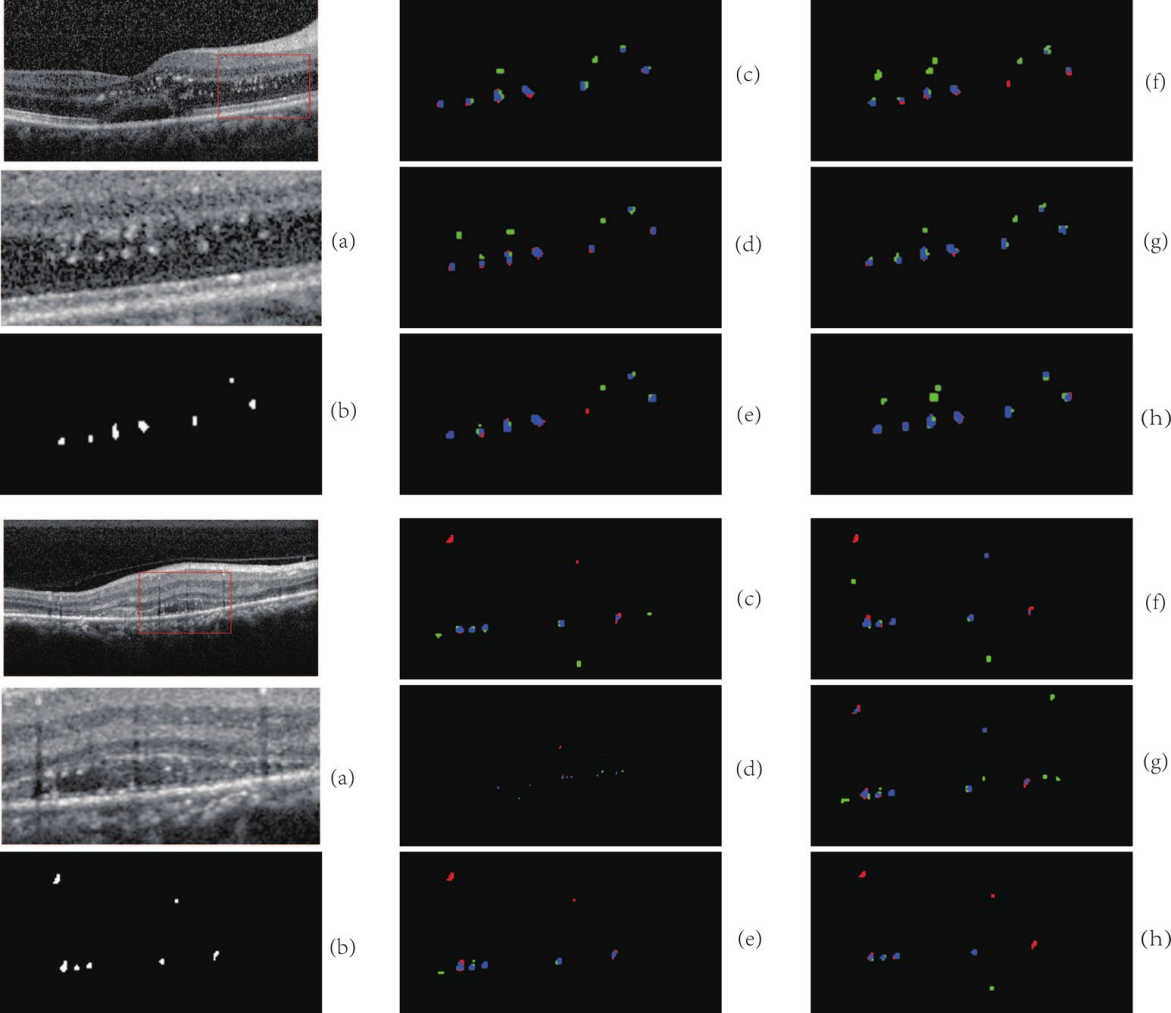
The locally enlarged images of the segmentation. Blue is TP, green is FP, red is FN. **a** are original images. **b** are enlarged original images. **c** are images of U-Net. **d** are images of U-Net++. **e** are images of Attention U-Net. **f** are images of SANet. **g** are images of our model

The same experimental environment and data set are used for training and tested on the same test set. The obtained segmentation results are shown in Fig. 8 and the locally enlarged images of the segmentation results are shown in Fig. 9.

We can observe from the figures that all of these networks have missing and erroneous segmentation. U-Net has more obvious missing segmentation, but there are few cases of missegmentation. U-Net++ integrates different features by connecting U-Net of different levels together, improving the P but having the same problem with U-Net. And the Attention U-Net can make the network learn the target area that needs to pay attention to when skipping connections and increase the weight of the target area, making the missing segmentation for HRF greatly reduced. But this network also erroneously focus on some background information areas, and identify the background information as HRF, leading to more erroneous segmentation. The SANet network incorporates an adaptive module in the last layer of the network so that the senior semantic information in the deep layer gain more attention, and the suppression of irrelevant information is more effective. However, it pays insufficient attention to the target region, resulting in a higher rate of missing segmentation with fewer errors. Our algorithm pays more attention to detailed information. In terms of TransUNet, transformer’s attention mechanism excels at capturing global contextual information, which contributes to increased sensitivity. However, this global focus can also lead to the model being influenced by irrelevant features, causing it to trigger positive predictions in non-lesion areas as well, thus reducing accuracy. Therefore, compared our model with U-Net, the missing segmentation is greatly reduced and erroneous segmentation is acceptable, leading to a better effectiveness of segmentation.

**Table 2:** Comparison of evaluation index among different networks

Network architecture	HRF region			HRF number		
	SE	P	DSC	SE	P	DSC
U-Net	58.44	68.84	60.32	64.92	68.88	65.78
U-Net++	61.48	70.66	61.22	62.26	72.08	63.55
Attention U-Net	75.74	60.28	65.02	78.67	64.34	68.62
SANet	56.58	70.04	62.58	60.64	73.06	64.34
TransUNet	71.88	64.08	65.44	74.02	70.54	69.18
Our model	72.90	66.12	66.84	73.57	70.70	71.32

Table 2 shows the comparison of evaluation index among different networks. According to the evaluation index, U-Net is less sensitive but more accurate, indicating that although U-Net can precisely identify some HRF, but the missing segmentation is severe. Compared with U-Net, the SE and P of U-Net++ segmentation are ameliorated. However, SE of U-Net++ is still relatively low, indicating that missing segmentation is still a problem. The same question as U-Net, SANet has improved P but greatly decreased SE. After training, the SE of Attention U-Net is 75.74$\%$, and P is only 60.28$\%$. Compared with U-Net, SE of Attention U-Net is greatly improved, but P is also decreased, indicating that the Attention module pay attention to details related to the target. At the same time, some irrelevant information is also focused on, resulting in an obvious over-segmentation phenomenon. In terms of reasons, on the one hand, relying on the network structure described in the paper to reproduce without open source code may have some errors. On the other hand, it is observed that the manual annotation of HRF not only includes HRF but also includes HE, which is different from the HRF defined in this paper. Because the adaptive module is only added to the deep layer, the network increased the suppression of irrelevant regions, leading to the improvement of P. However, the missing segmentation problem is not settled. Both TransUNet and Attention U-Net exhibit high sensitivity and lower accuracy. The attention mechanism, whether it’s the Transformer in TransUNet or the attention module in Attention U-Net, excels at capturing global contextual information. This contributes to increased sensitivity, as it allows the integration of information from different image regions to identify the target. However, this global focus can also lead to the model being influenced by irrelevant features, causing it to trigger positive predictions in non-target areas, thus reducing accuracy.

The algorithm in this paper focuses on more detailed information, resulting in slightly decreased P but an remarkable increase of SE. To sum up, the effectiveness of comprehensive segmentation is better than the other four networks. Except for it, because the number of network layers in this paper is few, and the number of channels in each layer is significantly fewer than that of U-Net, model parameters are greatly reduced.

### Ameliorated KiU-Net performs better than ameliorated U-Net

In addition to the comparison with different network models, this paper also reduce the number of U-Net layers to three layers for experiments, namely, only two downsamplings in the encoder stage.

**Fig. 10:**
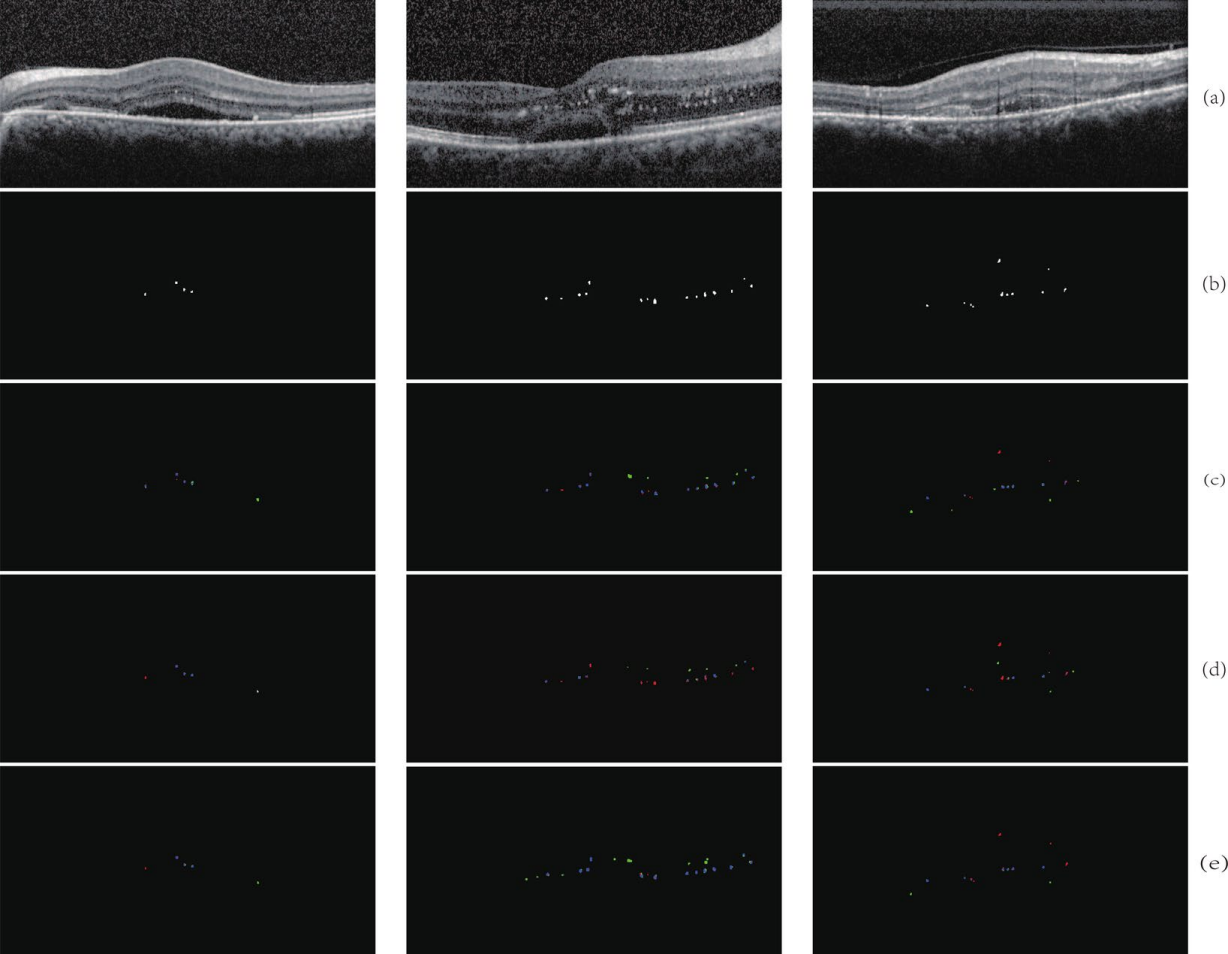
Comparison with three-layer U-Net segmentation results (blue is TP, green is FP, red is FN). **a** are original images. **b** are images of U-Net. **c** are images of U-Net with three layers. **d** are images of our model

**Fig. 11:**
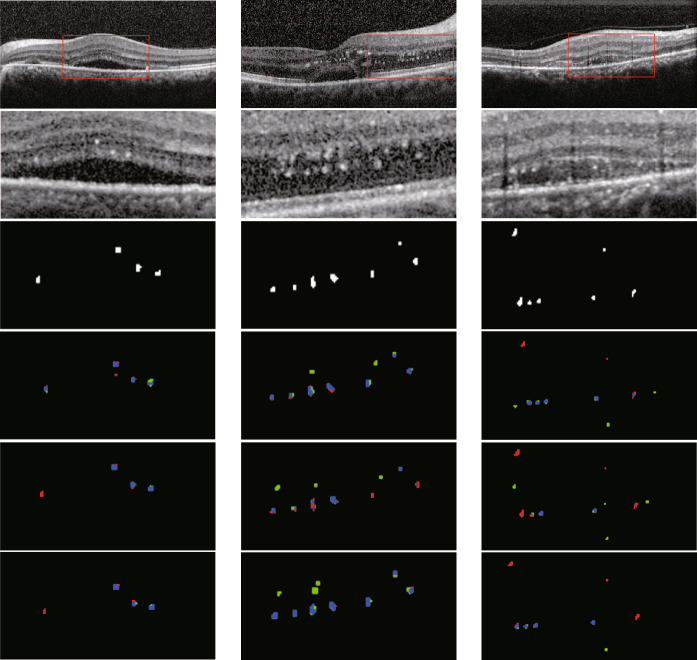
Comparison with three-layer U-Net segmentation results (blue is TP, green is FP, red is FN, and black is TN). **a** are original images. **b** are images of U-Net. **c** are images of U-Net with three layers. **d** are images of our model

Figure 10 presents a comparison of the segmentation results obtained using a three-layer U-Net and our proposed model. Figure 11 provides a magnified view of the comparative results. It can be observed that, compared to the three-layer U-Net, reducing the number of network layers eliminates the interference of high-level semantic information during feature extraction. Consequently, the network focuses more on shallow details, leading to an improvement in sensitivity (SE) and a significant enhancement in overall performance.

Table 3 shows the calculation of the evaluation index, and the comparison of evaluation index with U-Net of three layers.

**Table 3:** the comparison of evaluation index with U-Net of three layers

Network architecture	HRF region			HRF number		
	SE	P	DSC	SE	P	DSC
U-Net	58.44	68.84	60.32	64.92	68.88	65.78
Three-layer U-Net	61.78	68.44	61.85	65.30	68.76	66.20
Our model	72.90	66.12	66.84	73.57	70.70	71.32

In general, the results of experiments show that the addition of fine-scale information can ameliorate the problem of HRF missing segmentation and greatly improve SE. However, due to the lack of senior semantic information extracted from the deep layer of U-Net, the network would not recognize the background accurately, making some pixels belonging to the background be identified as targets and decreases the P. Therefore, compared with other networks, the algorithm of our model improves the performance of HRF segmentation.

## Discussion

The pathogenesis of DR, which contains basal layer thickening, the proliferation of endothelial cells, and the selective loss of pericytes, is elaborate and not completely illuminated. At present, the damage caused by DR may be irreversible, with no or slight symptoms at the early stage and a diminution of vision as the disease goes on. In the end, it may cause blindness. We now divide the DR into 6 stages, of which the first 3 stages belong to non-proliferative diabetic retinopathy(NPDR), without the generation of neovascularization. During this stage, capillaries can become slightly clogged, leaky, and bleeding. Morevoer, microaneurysms, small hemorrhagic spots, and exudation will appear on the retina due to being absorbed in the chronic hyperglycemic environment. The last 3 stages with apparent neovascularization, are proliferative diabetic retinopathy(PDR). At this stage, for being severely blocked, the leakage and bleeding of capillary causes retinal cells immersed in ischemia hypoxia. To maintain the retina blood supply, neovascularization and fiber hyperplasia begin to form on the optic disc and retina, which are easy to bleed and invade the vitreous body, and eventually cause tractional detachment of the retina [37].

If patients with diabetes can be screened regularly and diagnosed as early as possible, effective treatment can delay the development of the disease and avoid blindness [4]. DR patients also need regular follow-ups after diagnosis and treatment. OCT uses near-infrared light as the light source and uses the reflected infrared laser wave reflected from the tissue to form the image, with the characteristics of non-invasive, non-contact, and high resolution [38]. The microscopic structure of the retina and microscopic lesions in the fundus can be clearly observed and measured by OCT. Through the use of digital instruments, medical imaging segmentation can analyze pathological tissues quantitatively and qualitatively, and reproduce the 3D model of the tissues that doctors need [39]. How to segment medical image data with a balance between the recall ratio and recall P, and optimize the effectiveness is crucial to image segmentation, which helps to judge and evaluate the treatment effectiveness and prognosis of patients with DR [40]. Considering the problem of the small focal area and dispersed location of HRF, came up with an algorithm for HRF segmentation. On the one hand, the over-complete Kite-Net is used to increase the extraction of details in the process of feature extraction, so that the network pays more attention to the subtle structure of HRF. On the other hand, to adapt to the server performance and reduce the computational cost, only two layers of Kite-Net are used to complement the deep features of U-Net. In addition, a new cross-attention module is designed to integrate the features extracted from the Kite-Net branch and the U-Net branch to improve the feature representation ability of the two network branches. Experimental results show that the Kite-Net branch can improve the SE of HRF segmentation, but the P is decreased compared with the original U-Net. However, considering the SE and P of network segmentation, the segmentation effectiveness of HRF based on the ameliorated KiU-Net is still improved, and DSC is increased by 2$\%$ compared with other networks, indicating a better segmentation effectiveness. Moreover, the number of parameters of this model is greatly reduced, with only a few model parameters being used to achieve better segmentation accuracy.

However, the research on HRF segmentation has just begun, and there are still limitations in this research. The data obtained in this paper is still limited, and the problem of overfitting always exists in the process of network training. Although the overfitting has been alleviated by data enhancement, the enhanced data are highly similar, which is difficult to supplement richer feature information. All the experiments in this paper are trained and tested on the dataset established by ourselves, and the similarity between the training set and the test set is not excluded which may make the evaluation index of the test better. Therefore, more images can be added in the subsequent research, and we should consider using OCT imaging equipment from different manufacturers to acquire images and conduct joint training to enrich the dataset and ensure that the network can be fully trained. Due to the hard HE and HRF in OCT images performing similarly, we can consider establishing a joint segmentation algorithm. Or we can set up a system to classify them after the segmentation, according to the different sizes and back shadows, which further improves the accuracy of the segmentation of HRF. In addition to the difference between HE and HRF in OCT images, HE can be observed in fundus photography, but HRF cannot. Therefore, in the following research, we can establish multi-modal segmentation between OCT and fundus photography, judging the location of lesions segmented from OCT on fundus photography, to exclude the impact of HE on HRF, which helps improve the accuracy of HRF segmentation.

## Conclusion

With the further study of DR, researchers have found the existence of hyperreflective spots in OCT images of DR patients, and many studies have confirmed that the number of HRF is positively correlated with the development of DR. At present, many doctors regard the number of HRF as an important biomarker of the therapeutic effectiveness and prognosis of DR. In this paper, we proposed an improved KiU-Net segmentation algorithm to improve the effectiveness of the HRF segmentation. Using U-Net alone for segmentation usually ignore this tiny structure, by virtue of the small lesions of HRF in OCT images. KiU-Net is composed of Kite-Net branch and U-Net branch. Kite-Net branch replaces the downsampling of U-Net with upsampling coding, making the network obtain more detailed information and figure out the problem of U-Net that it cannot completely segment the tiny structure. Three layers of U-Net branch can extract high-level semantic information. In addition, we designed a new CAB to combine the information extracted from two branches, to further improve the feature representation of single branch network training. Results of experiments show that this model decreases the parameters and increase SE and DSC, providing a certain significance for the early detection, the evaluation of the treatment effectiveness, and the prognosis of patients with DR.
